# The original hotelling model with linear and quadratic firms logistics costs

**DOI:** 10.1371/journal.pone.0275197

**Published:** 2022-10-03

**Authors:** Mohammed Kharbach, Tarik Chfadi

**Affiliations:** 1 Africa Business School, Mohammed VI Polytechnic University, Ben Guerir, Morocco; 2 International Water Research Institute, Mohammed VI Polytechnic University, Ben Guerir, Morocco; Universitat Jaume I, SPAIN

## Abstract

We introduce a modified Hotelling model setup that incorporates the original model and considers logistics’ related costs incurred by the firms. Both the linear and quadratic logistics related costs cases are studied. We find that the relative magnitudes of firms’ logistics costs to customers’ transportation and/or search costs have significant effects on firms’ decisions in terms of location choices. In the linear case, we show, under a large set of cost structures, that substantial differentiation exists and is stable. In the quadratic case, maximum differentiation is the only equilibrium for all cost structures in the model but firms’ profits are higher than in the original case. We also consider the case where customers transportation costs are quadratic while firms logistics costs are linear.

## I. Introduction

Two of the most important works on location theory are the linear city model by Hotelling [1] and the work of d’Aspremont et al. [2]. The first work established the principle of minimum differentiation whereby, if two symmetric firms compete in a linear city and where consumers pay linear transportations costs, then both firms tend to locate in the middle of the consumers’ population line. The second work, using quadratic transportations costs and the same setup as the original work, introduced the principle of maximum differentiation by showing that the equilibrium in Hotelling’s original model is not stable when firms choose their locations strategically; firms tend to locate at the extreme points of the consumers’ population line instead.

There is a vast literature that discusses extensions of the Hotelling’s original model. Brenner [3] provides a survey about Hotelling literature on product differentiation, highlighting the determinants of equilibrium existence and the reasons for minimum and maximum differentiation outcomes. Many contributions have examined the effects of costs structures and customers’ distributions on equilibria outcomes. In a setup with an arbitrary number of players and for different customers’ distributions, Eaton and Lipsey [4] show that strong assumption are required for the principle of minimum differentiation to hold. Using the customers’ heterogeneity assumption, De Palma, Ginsburgh, Papageorgiou, and Thisse [5] show that Hotelling’s principle of minimum differentiation holds. Economides [6] considers the effects of reservation prices. Economides [7] considers the effects of transport costs. Meagher and Zauner [8] assess outcomes with different distributions of consumers. There are other categories of extensions of the original Hotelling model and chief among them are the consideration of space and dimensions. Irmen and Thisse [9] consider competition in multi-characteristics spaces and Chen and Riordan [10] use the spokes model for oligopolistic analysis. Lederer and Hurter [11] consider different firms and non-uniform distribution of consumers on the plane. Soetevent [12] extends Hotelling’s model of price competition with quadratic transportation costs from a line to graphs.

In Hotelling’s original model, consumers pay transportation costs and the competing firms charge a mill price. In reality, in most “shopping experiences”, both consumers and suppliers may incur some logistics-related costs. Indeed, consumers might bear only search costs while firms would incur the transportation costs; a consumer might visit a “shopping” center, select the product, and have the supplying firm deliver it to her home. This is also the case with online buying. This trend is likely to continue because of the digitalization of the economy and the “smartness” of urban development.

On the other hand, consumers might bear both search and transportation costs, while suppliers incur only some support costs. This is the case, for example, if firms are responsible for support services such as assembling and commissioning at customers’ locations but are not responsible for delivery. Another example is support services for remote installation and configuration of IT products.

From the examples above, it is evident that in many “shopping experiences”, firms do incur logistics-related costs that can be either for support-only costs, for transportation only costs, or for both support and transportation services costs. From the customers’ perspective, the costs could be either transportation costs (as in Hotelling’s original model), search costs (as in online buying) or both transportation and search costs.

Our main contribution is to model all of these logistical arrangements, from both the firms and consumers’ perspectives, in the same modified Hotelling framework. The original Hotelling model would be a special case of this modified setup. Our aim is to revisit the equilibrium stability issue of Hotelling’s original model with linear transportation costs, after introducing linear logistics related costs incurred by firms. We find that the logistics costs influence the firms’ decisions related to their locations choices. The Hotelling model with quadratic transportation costs for firms is also revisited using quadratic logistics costs.

Another contribution is the emphasis on the nature and the magnitude of consumers’ transportation costs (and obviously the nature and magnitude firms logistics costs), which is an overlooked element in Hotelling model literature. On the consumers’ side, we consider the costs paid, by consumers, above the product price, in a more disaggregated way. Indeed, such costs could be only search costs, when buying online for example. They are likely to be lesser in magnitude in such a case. For transportation costs (potentially including search costs) as in the original Hotelling model, it is reasonable to assume they are higher.

On the suppliers’ side, we introduce the logistics-related costs incurred by firms. Such costs may be lower in magnitude if there are no deliveries (only support costs) or higher if deliveries are involved. The relative magnitudes of firms’ logistics costs compared to customers’ transportation and/or search costs have important effects on firms’ decisions in terms of locations choices.

The remaining of this article is as follows. Section III is a description of the model with linear customers transportation costs linear logistics costs. Section IV considers both transportation and logistics quadratic costs and section V considers the mixed case where transportation costs are quadratic and logistics costs are linear. Section VI concludes.

## II. The linear model

We consider two firms A and B competing to supply a product to a population of consumers, which is uniformly distributed along the line [0,1] with a density equal to 1. A and B are located within the line [0,1] at points a and b respectively, such that:

0≤a≤b≤1

Our model differs from Hotelling’s original model in that both firms incur delivery and/or support costs in addition to production and other costs. In Hotelling’s original model, only the customers pay the transportation costs. In our model, these costs, can be interpreted as search costs or search and transportation costs depending on their levels relative to the costs paid by suppliers.

We consider identical firms and as in Hotelling’s original model and we are interested in symmetric equilibria. Our main aim is to see how the logistics-related costs incurred by suppliers and introduced here influence firms’ decisions and how the results compare to the ones in Hotelling’s original model. In particular, we are interested in whether there is a symmetric equilibrium in Hotelling’s original model framework, to which we have added the linear delivery and/or support costs.

We consider a two-stage game. In the first stage, both firms choose their locations (a and b). In the second stage, the two firms set their prices. The model is solved by backward induction. In the second stage, firm A (B) sets its price P_A_ (P_B_) to maximize its profit П_A_ (П_B_).

Production costs are equal to zero and the customer reservation price for the product is very high, which ensures that all consumers will be buy regardless of the product price.

The logistics-related cost function for firm A is piecewise linear and is defined as follows:

$$CA(x)={\{}_{t(x-a)if\;a\leq x\leq 1}^{t(a-x)if\;0\leq x\leq a},\;where\;t\;is\;the\;unit\;logistics\;cost.$$

### 2.1.Solving for the price equilibrium

In the second stage, firm A (B) sets its price P_A_ (P_B_) to maximize its profit П_A_ (П_B_). To derive the demand for each firm, we define the indifferent consumer by its location *x*_*I*_ as the consumer that would be indifferent in buying from firm A or B.

If *t*_*c*_ is the unit consumer transportation and/or search cot, then we have:

$$P_{A}+t_{c}(x_{I}-a)=P_{B}+t_{c}(b-x_{I})\Leftrightarrow x_{I}=\frac{P_{B}-P_{A}}{2t_{c}}+\frac{a+b}{2}$$

The firms’ profits are:

$$\Pi_{A}=P_{A}x_{I}-t\int_{0}^{a}(a-x)dx-t\int_{a}^{x_{I}}(x-a)dx$$

$$\Pi_{B}=P_{B}{(1-x}_{I})-t\int_{x_{I}}^{b}(b-x)dx-t\int_{b}^{1}(x-b)dx$$

Let $\alpha =\frac{t}{2t_{c}}$. Notice that if α is a high number, we have a situation that approximates the case of an integrated firm incurring delivery costs and consumers paying lower search costs. On the other hand, smaller values of α, are rather cases where consumers pay higher transportation (including search) costs.

Solving the FOCs (Appendix.A1 in S1 Appendix), we obtain,

$$-P_{A}[2+\alpha ]P_{B}[1+\alpha ]=t_{c}\{a(\alpha -1)-b(\alpha +1)\}$$ (1)

$$-P_{B}[2+\alpha ]{+P}_{A}[1+\alpha ]=t_{c}\{a(\alpha +1)-b(\alpha -1)-2\}$$ (2)

It is interesting to notice that the slope of the best reaction function of, say firm A is:

$$\frac{\partial P_{A}}{\partial P_{B}}=\frac{1+\alpha }{2+\alpha }=\frac{1}{2}(1+\frac{\alpha }{2+\alpha })$$

It is increasing from ½, which is the case in Hotelling’s original model, and converges asymptotically to one. This would indicate that as α increases, competition/differentiation becomes even stronger. Alternatively, this also indicates that when customers’ transportation and/or search costs are lower than logistics costs, which is the case with online shopping for example, competition becomes fiercer as the price dispersion discovery process is less costly.

Solving the system of Eqs (1) and (2), we get,

$$P_{A}=\frac{t_{c}}{3+2\alpha }\{b(2\alpha^{2}+3\alpha +1)-a(2\alpha^{2}+3\alpha -1)+2(1+\alpha )\}$$ (3)

$$P_{B}=\frac{t_{c}}{3+2\alpha }\{b(2\alpha^{2}+3\alpha -1)-a(2\alpha^{2}+3\alpha +1)+2(2+\alpha )\}$$ (4)

$$x_{I}=\frac{1}{6+4\alpha }\{2+(1+2\alpha )(a+b)\}$$ (5)

Notice that when α = 0, the results from Eqs (3)–(5) are similar to those from Hotelling’s original model, with linear transportation costs and no logistics costs.

### 2.2.Solving for the location equilibrium

In the first stage, the two companies choose their positions a and b to maximize their profits. The FOCs for the positions (Appendix.A.2 in S1 Appendix) yield:

$$\frac{1}{t_{c}}\frac{\partial \Pi_{A}}{\partial a}=-a\left\{\frac{28\alpha^{3}+84\alpha^{2}+63\alpha -2}{{2(3+2\alpha )}^{2}}\right\}+b\left\{\frac{4\alpha^{3}+12\alpha^{2}+9\alpha +2}{{2(3+2\alpha )}^{2}}\right\}+\left\{\frac{4\alpha^{2}+10\alpha +4}{{2(3+2\alpha )}^{2}}\right\}$$ (6)

$$\frac{1}{t_{c}}\frac{\partial \Pi_{B}}{\partial b}=a\left\{\frac{4\alpha^{3}+12\alpha^{2}+9\alpha +2}{{2(3+2\alpha )}^{2}}\right\}-b\left\{\frac{28\alpha^{3}+84\alpha^{2}+63\alpha -2}{{2(3+2\alpha )}^{2}}\right\}+\left\{\frac{-8+44\alpha +68\alpha^{2}+24\alpha^{3}}{{2(3+2\alpha )}^{2}}\right\}$$

In the following, we will define the conditions under which the profit function is concave (proposition.1), then we will narrow down the range to the values of α for which the symmetric optimal solution is an interior point (proposition.2) and finally we check that the profit functions are positive (proposition.3).

**Proposition.1**:

- The profit function is concave for α>α_0_, where α_0_ ≃0.03049.
- For α≤α_0_, the profit function is increasing in the position a. The two firms would locate at the middle of the line.

*Proof*. *(Appendix*.*A*.*3* in S1 Appendix*)*

We introduced some realistic features of the “shopping experience” to Hotelling’s original model, while preserving the linear costs that were responsible for the non-stability of the equilibrium. In contrast to Hotelling’s original model, the profit function is concave for a wide range of ratios of firms’ logistics costs and consumers’ transportation and/or search costs. This range includes both smaller and larger values, accounting for cases where integrated firms, for example, are responsible for the transportation while customers pay only smaller search costs, and cases where customers pay the higher transportation costs while firms pay smaller support costs.

On the other hand, the Principle of Minimum Differentiation applies for values of *α* in] 0, α_0_], extending the (non-stable) equilibrium results in the original Hotelling model. This range of cost ratios is rather closer to the case where consumers payi transportation costs while firms bear some support costs.

Proposition.2. defines the conditions for interior solutions.

**Proposition.2**:

- The interior symmetric location in stage one of the game is an interior solution in the semi-open interval] 0,1/2] for α≥ α_1_ ≃0.237≥ α_0_.
- The values of the position a in the interval] *α*_1_, 1/2] given by Eq (7) are interior solutions and are Nash equilibria in the prices and locations subgames if and only if 2<*α*.
- The Nash Equilibrium symmetric solutions interval is] 1/8, 10/56[, for firm A and] 46/56, 7/8 [for firm B.

*Proof*. *(Appendix*.*A*.*4* in S1 Appendix*)*

Contrary to Hotelling’s original model with a linear transportation cost, Proposition.1 shows that an equilibrium solution exists in this case. It is required to verify that the optimal solution is an interior one.

Indeed, the symmetric equilibrium is such that (Appendix.4 in S1 Appendix):

$$a=\frac{1}{8}\left(1+\frac{\left(2\alpha +2)(2\alpha +3\right)}{\alpha {\left(2\alpha +3\right)}^{2}}\right)=\frac{1}{8}\left(1+\frac{\left(2\alpha +2\right)}{\alpha (2\alpha +3)}\right)$$ (7)

$$b=\frac{-2+19\alpha +14\alpha^{2}}{8\alpha (3+2\alpha )}=\frac{1}{8}\left(7-\frac{2+2\alpha }{\alpha (3+2\alpha )}\right)$$

We have interior solutions for all α≥ α_1_ ≃0.237. Notice that the interior solutions satisfy the concavity condition α ≥ α_0._

For α_1_ > α≥ α_0,_ the profit function is concave and is increasing on the segment [0,1/2] but the optimal solution is larger than 1/2. As such, the firms would locate at the middle of the line.

To ensure that the interior solutions are Nash equilibria in the prices and the locations subgames, Appendix A.4.ii in S1Appendix shows the conditions under which no firm has incentives to undercut its rival by reducing its price and serving the whole market. We find that the values of the position a in the interval] *α*_1_, 1/2] given by Eq (7) are interior solutions and are Nash equilibria in the prices and locations subgames if and only if 2<*α*. As such, for firm A, the Nash Equilibrium symmetric solutions interval is] 1/8, 10/56 [and] 46/56, 7/8 [for firm B.

Proposition.3 checks the feasibility of the equilibria derived in Proposition.2 by verifying that the corresponding profits are positive.

**Proposition.3**.

The profits of the firms are positive for all α>0.

*Proof*.*(Appendix*.*A*.*5* in S1 Appendix*)*.

In this section, Hotelling’s original model is extended by considering the logistics-related costs that suppliers might incur. We focus on symmetric equilibria to preserve the main features of Hotelling’s original model for comparison purposes. Logistics-related costs are captured through the ratio of delivery and/or support costs to the customer’s search and/or transportation cost. We find that the results in Hotelling’s original model with linear transportation costs can be extended to cases where the supplying firms incur lower delivery and/or support costs (firms locating at ½). Most importantly, for medium and higher delivery costs, symmetric equilibria are found and firms tend to locate, asymptotically, at (1/8 and 7/8) of the market line. Looking at the distance to the location of selling firms as consumers’ perception of the quality offered by firms, the modified model shows that the customization of firms’ offers translates into an increasing differentiation of those offers.

## III. The quadratic model

In the quadratic model, we consider the same setup in the previous sections, namely two firms A and B competing to supply a uniform distribution of consumers along the line [0, 1] and located at points a and b such that 0≤*a*≤*b*≤1. We also consider a similar two-stage game, where a firm decides on its location a (b) in the first stage, then sets the price P_Aq_ (P_Bq_) to maximize its profits П_Aq_ (П_Bq_).

We keep the same assumptions regarding production costs and customer’s reservation price. However, in the present case, the costs are quadratic with respect to distance. The logistics-related cost function for firm A is now defined as:

$$C_{Aq}(x)=t{\left(x-a\right)}^{2}\;for\;all\;0\leq x\leq 1$$

### 3.1.Solving for the price equilibrium

In the second stage, and similar to the linear model, firm A (respectively Firm B) sets its price P_Aq_ (respectively P_Bq_) to maximize its quadratic profit *П*_*Aq*_ (respectively *П*_*Bq*_). Here again, we solve for the location of the independent consumer *x*_*Iq*_, who is indifferent between buying from firm A or B, which will help derive the demand for each firm. The location of consumer *x*_*Iq*_ is such that:

$$P_{Aq}+t_{c}{\left(x_{Iq}-a\right)}^{2}=P_{Bq}+t_{c}{\left({b-x}_{Iq}\right)}^{2}\Leftrightarrow x_{Iq}=\frac{P_{Bq}-P_{Aq}}{2(b-a)t_{c}}+\frac{a+b}{2}$$

With quadratic costs, the profits of the firms are:

$$\Pi_{Aq}=P_{Aq}x_{Iq}-t\int_{0}^{x_{Iq}}{\left(x-a\right)}^{2}dx$$

$$\Pi_{Bq}=P_{Bq}{(1-x}_{Iq})-t\int_{x_{Iq}}^{1}{\left(b-x\right)}^{2}dx$$

Given $\alpha =\frac{t}{2t_{c}}$, solving for the FOCs in P_Aq_ and P_Bq_ (Appendix.B.1 in S1 Appendix), we get:

$$\frac{\alpha (b-a)}{4}+\frac{a+b}{2}=P_{Aq}\frac{\alpha (\alpha +2)}{(b-a)t}-P_{Bq}\frac{\alpha (\alpha +1)}{(b-a)t}-{\left(P_{Bq}-P_{Aq}\right)}^{2}\frac{\alpha^{3}}{{\left(b-a\right)}^{3}t^{2}}$$ (8)

$$\frac{\alpha (b-a)}{4}+\frac{2-a-b}{2}=-P_{Aq}\frac{\alpha (\alpha +1)}{(b-a)t}+P_{Bq}\frac{\alpha (\alpha +2)}{(b-a)t}-{\left(P_{Bq}-P_{Aq}\right)}^{2}\frac{\alpha^{3}}{{\left(b-a\right)}^{3}t^{2}}$$ (9)

With quadratic costs, the slope of the best reaction function of, say firm A, becomes:

$$\frac{\partial P_{Aq}}{\partial P_{Bq}}=\frac{\left(\alpha +1){\left(b-a\right)}^{2}t+\alpha^{2}(P_{Bq}-P_{Aq}\right)}{\left(\alpha +2){\left(b-a\right)}^{2}t+\alpha^{2}(P_{Bq}-P_{Aq}\right)}$$

It is interesting to notice that the same trends subsist as in the linear costs case, with the reaction (1/2) simulating the original Hotelling model at *a* = 0, and converging asymptotically to 1, to reflect more aggressive competition.

Solving the system of Eqs (8) and (9), we get,

$$P_{Aq}=\frac{t}{4\alpha {\left(3+2\alpha \right)}^{2}}\{4\alpha -4a(3+7\alpha +2\alpha^{2})+4b(3+3\alpha +2\alpha^{2})+a^{2}(-6+9\alpha +12\alpha^{2}+4\alpha^{3})-2ab(5\alpha +12\alpha^{2}+4\alpha^{3})+b^{2}(6+17\alpha +12\alpha^{2}+4\alpha^{3})\}$$ (10)

$$P_{Bq}=\frac{t}{4\alpha {\left(3+2\alpha \right)}^{2}}\{4\alpha -4a(6+9\alpha +2\alpha^{2})+4b(6+5\alpha +2\alpha^{2})+a^{2}(6+17\alpha +12\alpha^{2}+4\alpha^{3})-2ab(5\alpha +12\alpha^{2}+4\alpha^{3})+b^{2}(-6+9\alpha +12\alpha^{2}+4\alpha^{3})\}$$ (11)

$$x_{Iq}=\frac{1}{6+4\alpha }\{2+(1+2\alpha )(a+b)\}$$ (12)

Notice that while the firms’ prices, P_Aq_ and P_Bq_, are different from those derived from the equilibrium with linear costs, the location of the independent consumer is similar to the one in the linear case. Also, with quadratic transportation costs, the case with α = 0 would bring about the original Hotelling model.

### 3.2.Solving for the location equilibrium

As in the linear costs case, the two firms choose their positions, a and b, in the first stage, to maximize their profits. The FOCs for the positions (Appendix.B.2 in S1 Appendix) give:

$$\frac{\partial \Pi_{Aq}}{\partial a}=\frac{t}{4\alpha {\left(3+2\alpha \right)}^{2}}\{-4(3+4\alpha )-4a(6+21\alpha +20\alpha^{2}+4\alpha^{3})-8b(\alpha +2\alpha^{2})-a^{2}{\left(1+2\alpha \right)}^{2}(9+14\alpha +4\alpha^{2})-2ab(3+\alpha ){\left(1+2\alpha \right)}^{3}+3b^{2}{\left(1+2\alpha \right)}^{2}\}$$ (13)

$$\frac{\partial \Pi_{Bq}}{\partial b}=\frac{t}{4\alpha {\left(3+2\alpha \right)}^{2}}\left\{8(6+23\alpha +33\alpha^{2}+20\alpha^{3}+4\alpha^{4})-2a(11\alpha +38\alpha^{2}+36\alpha^{3}+8\alpha^{4})-2b(24+111\alpha +178\alpha^{2}+116\alpha^{3}+24\alpha^{4})-3a^{2}{\left(1+2\alpha \right)}^{2}+2ab(3+\alpha ){\left(1+2\alpha \right)}^{3}+b^{2}{\left(1+2\alpha \right)}^{2}(9+14\alpha +4\alpha^{2})\right\}$$ (14)

**Proposition.4**:

In the Hotelling model with quadratic logistics costs, firms A and B locate, respectively, at a = 0 and b = 1. In particular, we have the following results:

- The profit function *Π*_*Aq*_ reaches its maximum at a = 0;
- The profit function *Π*_*Bq*_ reaches its maximum at b = 1;
- The profit functions of firms A and B are positive at a = 0 and b = 1, respectively;
- The profits in this model are higher than in Hotelling’s original model without logistics costs.
- The equilibrium is Nash perfect in the prices and locations subgames if 0 ≤*α*<4

Proof.

- The second derivative of the profit function of firm A is negative for all 0≤*a*, (Appendix B.**3**.ii in S1 Appendix). As such, the first derivative is decreasing in a. The first derivative of firm A’s profit is negative at a = 0 (Appendix **B.**3.i in S1 Appendix). As such, it is negative for all 0≤*a*. Therefore, firm A’s profit function is maximal at a = 0 for interior solutions.
- Similarly, the second derivative of the profit function for firm B is negative in the unit interval (Appendix B.3.ii in S1 Appendix). As such, the first derivative is decreasing in b. The first derivative of firm B’s profit is positive at b = 1 (Appendix 3.i in S1 Appendix). As such, it is positive for all 0≤*b*≤1. Therefore, firm B’s profit function is maximal at b = 1 for interior solutions.
- The resulting prices are:
$$P_{Aq}=P_{Bq}=\frac{t(2+\alpha )}{4\alpha }=t_{c}(1+\frac{\alpha }{2})$$
  The independent consumer is located, as in the linear costs case, at the middle *x*_*Iq*_ = +1/2, and the profits are positive and given as:
$$\Pi_{Aq}=\Pi_{Bq}=\frac{t(3+\alpha )}{12\alpha }=t_{c}(\frac{1}{2}+\frac{\alpha }{6})$$
- In Hotelling’s original model with quadratic transportation costs and no logistics costs, the profit of say firm A is:
$$\Pi_{As}=\frac{t_{c}}{2}$$
  In the Hotelling model with quadratic logistics costs, a profit “premium” of $\frac{{\alpha t}_{c}}{6}=\frac{t}{12}$, is achieved. Note that once t equates zero, the profit premium vanishes since we are in the original setup of Hotelling model. It should be noted that one would expect profits in our modified Hotelling set up to be lower than in the original Hotelling model since firms are incurring additional costs. But as t increases, differentiation intensifies, leading to higher prices and profits. An alternative interpretation of the resulting premium is that efforts to customize the product to consumers are rewarded with higher prices.
- The interior solution in the quadratic case (a = 0, b = 1) is Nash equilibrium in the prices and locations subgames if 0<*α*<4.

In this section, Hotelling’s original model with quadratic costs is extended by considering quadratic logistics related costs that suppliers might incur. Logistics-related costs are captured through the ratio of delivery and/or support cost to customer search and/or transportation costs. We find that the results of Hotelling’s original model with quadratic transportation costs (maximum differentiation principle) hold in all cases where producing firms incur logistics-related costs. However, the profits achieved in the modified Hotelling model are higher than in the original model.

## IV. The Mixed model

In this case, we consider that firms have linear logistics costs and that customers have quadratic transportation costs.

### 4.1.Solving for the price equilibrium

In the second stage, and similarly to the linear model, firm A (respectively Firm B) sets its price P_Am_ (respectively P_Bm_) to maximize its mixed profit *П*_*Am*_ (respectively *П*_*Bm*_). Here again we solve for the location of the independent consumer *x*_*Im*_, indifferent between buying from firm A or B, which will help deriving the demand for each firm. The location of consumer *x*_*Im*_ is (similar to the quadratic model) such that:

$$P_{Am}+t_{c}{\left(x_{Im}-a\right)}^{2}=P_{Bm}+t_{c}{\left({b-x}_{Im}\right)}^{2}\Leftrightarrow x_{Im}=\frac{P_{Bm}-P_{Am}}{2(b-a)t_{c}}+\frac{a+b}{2}$$

With mixed cost structures, the profits of the firms are:

$$\Pi_{Am}=P_{Am}x_{Im}-t\int_{0}^{a}(a-x)dx-t\int_{a}^{x_{Im}}(x-a)dx$$

$$\Pi_{Bm}=P_{Bm}{(1-x}_{Im})-t\int_{x_{Im}}^{b}(b-x)dx-t\int_{b}^{1}(x-b)dx$$

Given $\alpha =\frac{t}{2t_{c}}$, solving the FOCs in P_Am_ and P_Bm_ (Appendix.C.1 in S1 Appendix), we get:

$$\frac{\alpha }{2}+\frac{a+b}{2}=-\frac{P_{Bm}-2P_{Am}}{2(b-a)t_{c}}-\alpha \left[\frac{P_{Bm}-P_{Am}}{{2(b-a)}^{2}t_{c}}\right]$$

$$\frac{\alpha }{2}+\frac{2-a-b}{2}=\frac{{2P}_{Bm}-P_{Am}}{2t_{c}(b-a)}+\alpha \left[\frac{P_{Bm}-P_{Am}}{{2t_{c}(b-a)}^{2}}\right]$$

Therefore, we get:

$$P_{Am}=\frac{(b-a)t_{c}}{\left(2\alpha +3b-3a\right)}\{\left(b-a)(2+3\alpha +a+b)+2\alpha (1+\alpha \right)\}$$ (15)

$$P_{Bm}=\frac{(b-a)t_{c}}{\left(2\alpha +3b-3a\right)}\{\left(b-a)(4+3\alpha -a-b)+2\alpha (1+\alpha \right)\}$$ (16)

$$x_{Im}=\frac{b(2+b+2\alpha )-a(2+a-2\alpha )}{\left(2\alpha +3b-3a\right)}$$ (17)

### 4.2.Solving for the location equilibrium

Similar to the case with linear costs, the two companies chose their positions, a and b, in the first stage, to maximize their profits.

**Proposition.5**.

- The interior solutions are defined as:
$$a=1-b=\frac{-1+3\alpha +2\alpha^{2}}{[1+17\;\alpha +8\;\alpha^{2}+\sqrt{9+58\;\alpha +145\;\alpha^{2}+176\;\alpha^{3}+64\;\alpha^{4}}}$$
- The position of firm A is positive and increasing for $\alpha >\alpha_{c}=\frac{1}{4}(-3+\sqrt{17})$. It converges to $\frac{1}{8}$ when *α* goes to infinity. Hence, it is an interior solution.
- The interior solutions defined above are Nash equilibria in prices and locations,

*Proof*, *Appendix*. *C* in S1 Appendix.

## V. Conclusion

We have provided an extension of the original Hotelling model for both the linear and quadratic costs cases. The only additional assumption introduced concerns the logistics-related costs incurred by firms. Indeed, most of the “shopping experiences”, in reality, involve some logistics costs to the suppliers. We focused on symmetric equilibria to preserve the main features of Hotelling’s original model for comparison purposes. The magnitude of customers and firms’ costs are captured through the ratio of the firms logistics costs to the customer search and/or transportation cost. We have considered three cases. In the first case, the customers have linear transportation costs while the firms have also linear logistics costs. In the second case, customers (firms) have quadratic transportation (logistics) costs. In the third case, customers (firms) have quadratic (linear) transportation (logistics) costs.

Our main result is that, as opposed to the principle of minimum differentiation and the non-stable equilibrium of Hotelling’s original model with linear transportation costs, substantial differentiation exists and is stable. Hotelling’s original model is a particular case of ours and we find that the principle of minimum differentiation holds when the firms’ logistics costs are lower compared to consumers’ transportation costs.

Looking at the distance to the location of selling firms’ as consumers’ perception of the quality offered by firms, the modified model shows that customization of firms’ offers translates into an increasing differentiation of such offers. A limitation of the model, when used to assess customization decision, is on the non-incorporation of customers reactions to such customization efforts. However, the same set up can be used to capture customers’ reactions to customization investments by allowing, for example, the customers disutility (*t*_*c*_) to be reduced to a lower value *(t*_*c*_*-βt)* following firms customization decision (*t*).

In the case of logistics related costs that are quadratic, the maximum differentiation principle holds and the achieved profits are unexpectedly higher than in Hotelling’s original model due of stronger competition.

Finally, in the mixed case, interior and stable solutions exist and the two firms tend to move inwards in the interval [0,1]. Firm A(B) position tends to 1/8 (7/8) when *α* goes to infinity.

## Supporting information

S1 Appendix (DOCX)
