# Supplementary material for: The original hotelling model with linear and quadratic firms logistics costs
Source: PLoS One. 2022 Oct 3;17(10):e0275197. doi: 10.1371/journal.pone.0275197 (PMC9529135; doi:10.1371/journal.pone.0275197)
Supplement: S1 Appendix — (DOCX) [file pone.0275197.s001.docx]

**Appendices.**

**Appendix A – The Linear Costs Model**

**Appendix A.1:**

Firm A’s profit is:

$\Pi_{A}=P_{A}x_{I}-t\int_{0}^{a} \left( a-x \right)dx-t\int_{a}^{x_{I}} (x-a)dx$=$P_{A}x_{I}-ta^{2}-t(\frac{{x_{I}}^{2}}{2}-ax_{I})$

$$\frac{\partial\Pi_{A}}{\partial P_{A}}=\frac{P_{B}-{2P}_{A}}{2t_{c}}+\frac{a+b}{2}-t\left[ x_{I}\left( \frac{-1}{2t_{c}} \right)+\frac{a}{2t_{c}} \right]=\frac{P_{B}-{2P}_{A}}{2t_{c}}+\frac{a+b}{2}+\frac{t}{{2t}_{c}}\left[ \frac{P_{B}-P_{A}}{2t_{c}}+\frac{b-a}{2} \right]$$

$$\frac{\partial\Pi_{A}}{\partial P_{A}}=0\Rightarrow\frac{P_{B}}{2t_{c}}\left[ 1+\frac{t}{2t_{c}} \right]-\frac{P_{A}}{2t_{c}}\left[ 2+\frac{t}{2t_{c}} \right]=\frac{a}{2}\left[ \frac{t}{2t_{c}}-1 \right]-\frac{b}{2}\left[ \frac{t}{2t_{c}}+1 \right]$$

Let $\alpha=\frac{t}{2t_{c}}$. We get:

$-P_{A}\left[ 2+\alpha\right]{+P}_{B}\left[ 1+\alpha\right]=t_{c}\{a(\alpha-1)-b(\alpha+1)\}$ (1)

$-P_{B}\left[ 2+\alpha\right]{+P}_{A}\left[ 1+\alpha\right]=t_{c}\{a\left( \alpha+1 \right)-b\left( \alpha-1 \right)-2\}$ (2)

**Appendix A.2:**

We have

$$\Pi_{A}=P_{A}x_{I}-ta^{2}-t\left( \frac{{x_{I}}^{2}}{2}-ax_{I} \right), so\frac{\Pi_{A}}{t_{c}}={\frac{1}{t_{c}}P}_{A}x_{I}-2\alpha\left( \frac{{x_{I}}^{2}}{2}-ax_{I}+a^{2} \right)$$

$$\frac{1}{t_{c}}\frac{\partial\Pi_{A}}{\partial a}=-\frac{2\alpha^{2}+3\alpha-1}{3+2\alpha}x_{I}+\frac{1+2\alpha}{6+4\alpha}\frac{P_{A}}{t_{c}}-2\alpha\left( \frac{1+2\alpha}{6+4\alpha}x_{I}-x_{I}-a\frac{1+2\alpha}{6+4\alpha}+2a \right)$$

$$\frac{1}{t_{c}}\frac{\partial\Pi_{A}}{\partial a}=\frac{1+2\alpha}{3+2\alpha}x_{I}+\frac{1+2\alpha}{6+4\alpha}\frac{P_{A}}{t_{c}}-2\alpha\frac{11+6\alpha}{6+4\alpha}a$$

$$So \frac{1}{t_{c}}\frac{\partial\Pi_{A}}{\partial a}=a\left\{ \frac{\left( 1+2\alpha\right)^{2}}{{2\left( 3+2\alpha\right)}^{2}}-\frac{\left( 1+2\alpha\right)\left( 2\alpha^{2}+3\alpha-1 \right)}{{2\left( 3+2\alpha\right)}^{2}}-\frac{2\alpha\left( 3+2\alpha\right)\left( 11+6\alpha\right)}{{2\left( 3+2\alpha\right)}^{2}} \right\}+b\left\{ \frac{\left( 1+2\alpha\right)^{2}}{{2\left( 3+2\alpha\right)}^{2}}+\frac{\left( 1+2\alpha\right)\left( 2\alpha^{2}+3\alpha+1 \right)}{{2\left( 3+2\alpha\right)}^{2}} \right\}+\left\{ \frac{2(1+2\alpha)}{{2\left( 3+2\alpha\right)}^{2}}+\frac{2\left( 1+2\alpha\right)\left( 1+\alpha\right)}{{2\left( 3+2\alpha\right)}^{2}} \right\}$$

$So \frac{1}{t_{c}}\frac{\partial\Pi_{A}}{\partial a}=-a\left\{ \frac{28\alpha^{3}+84\alpha^{2}+63\alpha-2}{{2\left( 3+2\alpha\right)}^{2}} \right\}+b\left\{ \frac{4\alpha^{3}+12\alpha^{2}+9\alpha+2}{{2\left( 3+2\alpha\right)}^{2}} \right\}+\left\{ \frac{4\alpha^{2}+10\alpha+4}{{2\left( 3+2\alpha\right)}^{2}} \right\}$ (6)

**Appendix A.3:**

1. The second derivative of firm A’s profit function for (Equation.6 in Appendix.2) is:

$$\frac{{\partial^{2}\Pi}_{A}}{\partial^{2}a}=-\left\{ \frac{28\alpha^{3}+84\alpha^{2}+63\alpha-2}{{2\left( 3+2\alpha\right)}^{2}} \right\}$$

Let $g\left( \alpha\right)=28\alpha^{3}+84\alpha^{2}+63\alpha-2$. The function g is increasing in α and is positive for α>α_0_, where α_0_ ≃0.03049$.$ So for α>α_0_, the profit function is concave.

1. For α≤α_0_,

$$-a\left\{ \frac{28\alpha^{3}+84\alpha^{2}+63\alpha-2}{{2\left( 3+2\alpha\right)}^{2}} \right\} is positive$$

Therefore, the first derivative (Equation.6 in Appendix A.2) is positive ensuring that the profit function is increasing in a.

**Appendix A.4:**

1. The first derivatives with respect to a and b yield,

$$\frac{\partial\Pi_{A}}{\partial a}=0\Rightarrow a\left( 28\alpha^{3}+84\alpha^{2}+63\alpha-2 \right)-b\left( 4\alpha^{3}+12\alpha^{2}+9\alpha+2 \right)=(4\alpha^{2}+10\alpha+4)$$

$\frac{\partial\Pi_{B}}{\partial b}=0\Rightarrow-b\left( 28\alpha^{3}+84\alpha^{2}+63\alpha-2 \right)+a\left( 4\alpha^{3}+12\alpha^{2}+9\alpha+2 \right)=(8-24\alpha^{3}-72\alpha^{2}-44\alpha)$.

For our symmetric set up, we obtain

$$a=1-b=\frac{1}{8}\frac{4\alpha^{3}+16\alpha^{2}+19\alpha+6}{4\alpha^{3}+12\alpha^{2}+9\alpha}=\frac{1}{8}\left( 1+\frac{4\alpha^{2}+10\alpha+6}{4\alpha^{3}+12\alpha^{2}+9\alpha} \right)$$

$$a=\frac{1}{8}\left( 1+\frac{\left( 2\alpha+2 \right)\left( 2\alpha+3 \right)}{\alpha\left( 2\alpha+3 \right)^{2}} \right)=\frac{1}{8}\left( 1+\frac{\left( 2\alpha+2 \right)}{\alpha\left( 2\alpha+3 \right)} \right) (7)$$

$So a\leq\frac{1}{2}\Leftrightarrow6\alpha^{2}+7\alpha-2\geq0$. $let h\left( \alpha\right)=6\alpha^{2}+7\alpha-2$

The function *h* is increasing in α and is strictly positive for α>α_1_, where α_1_ ≃0.237$.$ We note that α_1_>α_0,_ which ensures that indeed a is an optimal (second derivative negative) and an interior solution.

For α_0_≤α<α_1_, the firms would locate at the center of the line (concave profit in a and the optimal solution is larger than ½).

1. We need to check whether the solutions above are perfect Nash equilibria in prices and locations subgames. For this, we need to derive the conditions under which firm A for example does not have any incentive to undercut firm B and serves all the market. This is equivalent to checking whether the optimal profit of firm A ($\Pi_{A})$assuming no undercutting is higher than firm A profit ($\Pi_{Au})$with an undercutting price P_Amin_ and assuming firm B still charges it is optimal price in the game without undercutting from firm A. We have:

$$P_{Amin}=P_{B}-t_{c}(b-a)$$

Therefore, we need:

$$\Pi_{A}>\Pi_{Au}, or$$

$$P_{A}*X_{I}-ta^{2}-\frac{t}{8}+a\frac{t}{2}>\left( P_{B}-t_{c}\left( b-a \right) \right)*1-t\int_{0}^{a} \left( a-x \right)dx-t\int_{a}^{1} \left( x-a \right)dx$$

We check the inequality above for the cases of interior solutions. It is easy to check that for interior solutions (a+b=1), we have:

$P_{A}=P_{B}=t_{c}\left\{ \alpha\left( b-a \right)+1 \right\} and x_{I}=\frac{1}{2}$, and,

$$\Pi_{A}= \frac{t_{c}}{2}\left\{ \alpha\left( b-a \right)+1 \right\}-ta^{2}-\frac{t}{8}+\frac{t}{2}a=\frac{t_{c}}{2}\left\{ \alpha\left( 1-2a \right)+1 \right\}+\frac{t}{2}a(1-2a)-\frac{t}{8}$$

$$\Pi_{A}=\left( \alpha\frac{t_{c}}{2}+\frac{t}{2}a \right)\left( 1-2a \right)+\frac{t_{c}}{2}-\frac{t}{8}=\left( \alpha\frac{t_{c}}{2}+\alpha t_{c}a \right)\left( 1-2a \right)+\frac{t_{c}}{2}-\frac{\alpha t_{c}}{4}=\alpha\frac{t_{c}}{2}\left( 1+2a \right)\left( 1-2a \right)+\frac{t_{c}}{2}(1-\frac{\alpha}{2})$$

Therefore,

$$\Pi_{A}=\alpha\frac{t_{c}}{2}\left( 1-4a^{2} \right)+\frac{t_{c}}{2}\left( 1-\frac{\alpha}{2} \right)$$

$$\Pi_{A}=t_{c}(\frac{\alpha+2}{4}-2\alpha a^{2})$$

$$\Pi_{Au}=\left( P_{B}-t_{c}\left( b-a \right) \right)*1-t\int_{0}^{a} \left( a-x \right)dx-t\int_{a}^{1} \left( x-a \right)dx=\left( P_{B}-t_{c}\left( b-a \right) \right)-\frac{ta^{2}}{2}-\frac{t}{2}-\frac{ta^{2}}{2}+ta$$

$$\Pi_{Au}=t_{c}\left\{ \alpha\left( b-a \right)+1 \right\}-{(1-2a)(t}_{c}+\frac{t}{2})-ta^{2}=(1-2a)( \alpha t_{c}-t_{c}-\frac{t}{2})+t_{c}-ta^{2}$$

$$\Pi_{Au}=(1-2a)( \alpha t_{c}-t_{c}-\alpha t_{c})+t_{c}-2\alpha t_{c}a^{2}$$

Therefore

$$\Pi_{Au}=t_{c}(2a-2\alpha a^{2})$$

$${\Delta\Pi=\Pi}_{A}-\Pi_{Au}=t_{c}\left( \frac{\alpha+2}{4}-2\alpha a^{2}-2a+2\alpha a^{2} \right)=t_{c}\left( \frac{\alpha+2}{4}-2a \right)>0 iff a<\frac{\alpha+2}{8}$$

As such,

The values of the position a in the interval ] $\alpha_{1},$1/2] given by equation (7) are interior solutions and are Nash equilibria in the prices and locations subgames if and only if $2<\alpha$.

**Appendix A.5:**

1. We need to check whether the symmetric and interior solutions (α≥α_1_≃0.237) ensure positive profits for firms. For firm A, we have:

$${\frac{1}{t_{c}}\Pi}_{A}={\frac{1}{t_{c}}P}_{A}x_{I}-2\alpha a^{2}-2\alpha\left( \frac{{x_{I}}^{2}}{2}-ax_{I} \right)={\frac{1}{{2t}_{c}}P}_{A}-2\alpha a^{2}+\alpha a-\frac{\alpha}{4}$$

For a symmetric solution, we have:

$$b=1-a\Rightarrow P_{A}=\frac{t_{c}}{3+2\alpha}\{\left( 2\alpha^{2}+5\alpha+3 \right)-a\left( 4\alpha^{2}+6\alpha\right)\}$$

$${\frac{1}{t_{c}}\Pi}_{A}=\frac{2\alpha^{2}+5\alpha+3}{2\left( 3+2\alpha\right)}-\alpha a-2\alpha a^{2}+\alpha a-\frac{\alpha}{4}=\frac{\alpha\left( 2\alpha+3 \right)+\left( 2\alpha+3 \right)}{2\left( 3+2\alpha\right)}-\alpha a-2\alpha a^{2}+\alpha a-\frac{\alpha}{4}$$

$${\frac{1}{t_{c}}\Pi}_{A}=\frac{\alpha}{4}+\frac{1}{2}-2\alpha a^{2}=\frac{\alpha}{4}+\frac{1}{2}-\frac{1}{32}(\alpha+\frac{2\left( 2\alpha+2 \right)}{\left( 3+2\alpha\right)}+\frac{\left( 2\alpha+2 \right)^{2}}{\alpha\left( 3+2\alpha\right)^{2}})$$

$${\frac{1}{t_{c}}\Pi}_{A}=\frac{7\alpha}{32}+\frac{1}{8}\frac{\left( 7\alpha+11 \right)}{\left( 3+2\alpha\right)}-\frac{1}{8}\frac{\left( \alpha+1 \right)^{2}}{\alpha\left( 3+2\alpha\right)^{2}})=\frac{1}{32}\frac{28\alpha^{4}+14{0\alpha}^{3}+231\alpha^{2}+124\alpha-4}{\alpha\left( 3+2\alpha\right)^{2}}$$

$$let n\left( \alpha\right)=28\alpha^{4}+14{0\alpha}^{3}+231\alpha^{2}+124\alpha-4$$

The function $n\left( \alpha\right)$ is increasing in α and is positive for α greater than α_2_=0.03049.

We have α_0_ =α_2_≤α_1_

Firm A’s profit for the optimal and interior solutions is positive.

1. For 0<α≤α_0_,

The profit function is increasing in a. For a=0 and b=1, we have, ${\frac{1}{t_{c}}\Pi}_{A}={\frac{1}{t_{c}}P}_{a}\frac{1}{2}-\left( \frac{\alpha}{4} \right)=\frac{1+\alpha}{2}-\frac{\alpha}{4}\geq0$. Therefore the profit function is also positive for 0<α≤α_0_ .

1. For α_1_ > α≥ α_0,_

For α_1_ > α≥ α_0,_ the profit function is concave and is increasing on the [0,1/2] segment and the profit at a=0 and b=1 is positive.

**Appendix B – The Quadratic Costs Model**

**Appendix B.1.**

The profit of firm A is:

$$\Pi_{Aq}=P_{Aq}x_{Iq}-\int_{0}^{x_{I}q} t \left( a-x \right)^{2}dx=P_{Aq}x_{Iq}-t\left( \frac{\left( x_{Iq}-a \right)^{3}+a^{3}}{3} \right)$$

$$\frac{\partial\Pi_{Aq}}{\partial P_{Aq}}=\frac{P_{Bq}-P_{Aq}}{2 \left( b-a \right)t_{c}}+\frac{a+b}{2}+P_{Aq}\frac{-1}{2 \left( b-a \right)t_{c}}-t\frac{-1}{2 \left( b-a \right)t_{c}}\left( x_{Iq}-a \right)^{2}$$

$$\frac{\partial\Pi_{Aq}}{\partial P_{Aq}}=\frac{P_{Bq}-{2P}_{Aq}}{2 \left( b-a \right) t_{c}}+\frac{a+b}{2}+\frac{t}{{2 \left( b-a \right) t}_{c}}\left( \frac{P_{Bq}-P_{Aq}}{2(b-a)t_{c}}+\frac{b-a}{2} \right)^{2}$$

$$\frac{\partial\Pi_{Aq}}{\partial P_{Aq}}=\frac{\left( b-a \right) t}{8 t_{c}}+\frac{a+b}{2}-\frac{P_{Aq}\left( t+4 t_{c} \right)}{4 \left( b-a \right) {t_{c}}^{2}}+\frac{P_{Bq}\left( t+2 t_{c} \right)}{4 \left( b-a \right) {t_{c}}^{2}}-\frac{\left( P_{Bq}-P_{Aq} \right)^{2}t}{8 \left( b-a \right)^{3}{t_{c}}^{3}}$$

$$\frac{\partial\Pi_{Aq}}{\partial P_{Aq}}=\frac{\left( b-a \right)}{4}\left[ \frac{t}{2t_{c}} \right]+\frac{a+b}{2}-\frac{P_{Aq}}{\left( b-a \right) t}\left[ \frac{t^{2}+4tt_{c}}{4{t_{c}}^{2}} \right]+\frac{P_{Bq}}{\left( b-a \right) t}\left[ \frac{t^{2}+2tt_{c}}{4{t_{c}}^{2}} \right]-\frac{\left( P_{bq}-P_{Aq} \right)^{2}}{\left( b-a \right)^{3}t^{2}}\left[ \frac{t}{2t_{c}} \right]^{3}$$

With $\alpha=\frac{t}{2t_{c}}$, we rewrite as:

$$\frac{\partial\Pi_{Aq}}{\partial P_{Aq}}=\frac{\alpha\left( b-a \right)}{4}+\frac{a+b}{2}-P_{Aq}\frac{\alpha\left( \alpha+2 \right)}{\left( b-a \right) t}+P_{Bq}\frac{\alpha\left( \alpha+1 \right)}{\left( b-a \right) t}+\left( P_{Bq}-P_{Aq} \right)^{2}\frac{\alpha^{3}}{\left( b-a \right)^{3}t^{2}}$$

$\frac{\partial\Pi_{Aq}}{\partial P_{Aq}}=0\Rightarrow\frac{\alpha\left( b-a \right)}{4}+\frac{a+b}{2}=P_{Aq}\frac{\alpha\left( \alpha+2 \right)}{\left( b-a \right) t}-P_{Bq}\frac{\alpha\left( \alpha+1 \right)}{\left( b-a \right) t}-\left( P_{Bq}-P_{Aq} \right)^{2}\frac{\alpha^{3}}{\left( b-a \right)^{3}t^{2}}$ (8)

For firm B the profit is:

$$\Pi_{Bq}=P_{Bq}{(1-x}_{I})-\int_{x_{I}}^{1} {t (b-x)}^{2}dx=P_{Bq}{(1-x}_{Iq})-t \left( \frac{\left( 1-b \right)^{3}+\left( b-x_{Iq} \right)^{3}}{3} \right)$$

$$\frac{\partial\Pi_{Bq}}{\partial P_{Bq}}={(1-x}_{Iq})-\frac{P_{Bq}}{2 \left( b-a \right)t_{c}}+ \frac{t}{2 \left( b-a \right)t_{c}}{{(b-x}_{Iq})}^{2}$$

$$\frac{\partial\Pi_{Bq}}{\partial P_{Bq}}=\left( 1-\frac{P_{Bq}-P_{Aq}}{2 \left( b-a \right)t_{c}}-\frac{a+b}{2} \right)-\frac{P_{Bq}}{2 \left( b-a \right)t_{c}}+ \frac{t}{2 \left( b-a \right)t_{c}}\left( b-\frac{P_{Bq}-P_{Aq}}{2 \left( b-a \right)t_{c}}-\frac{a+b}{2} \right)^{2}$$

$$\frac{\partial\Pi_{Bq}}{\partial P_{Bq}}=\frac{2-a-b}{2}+\frac{P_{Aq}{-2 P}_{Bq}}{2 \left( b-a \right)t_{c}}+ \frac{t}{2 \left( b-a \right)t_{c}}\left( \frac{P_{Bq}-P_{Aq}}{2 \left( b-a \right)t_{c}}-\frac{b-a}{2} \right)^{2}$$

$$\frac{\partial\Pi_{Bq}}{\partial P_{Bq}}=\frac{2-a-b}{2}+\frac{P_{Aq}{-2 P}_{Bq}}{2 \left( b-a \right)t_{c}}+\frac{\left( P_{Bq}-P_{Aq} \right)^{2}t}{8 \left( b-a \right)^{3}{t_{c}}^{3}}-\frac{t \left( P_{Bq}-P_{Aq} \right)}{4 \left( b-a \right) {t_{c}}^{2}}+\frac{\left( b-a \right) t}{8 t_{c}}$$

$$\frac{\partial\Pi_{Bq}}{\partial P_{Bq}}=\frac{2-a-b}{2}+\frac{\left( b-a \right) t}{8 t_{c}}+\frac{P_{aq}\left( t+2 t_{c} \right)}{4 \left( b-a \right) {t_{c}}^{2}}-\frac{P_{bq}\left( t+4 t_{c} \right)}{4 \left( b-a \right) {t_{c}}^{2}}+\frac{\left( P_{bq}-P_{aq} \right)^{2}t}{8 \left( b-a \right)^{3}{t_{c}}^{3}}$$

$$\frac{\partial\Pi_{Bq}}{\partial P_{Bq}}=\frac{2-a-b}{2}+\frac{\left( b-a \right)}{4}\left[ \frac{t}{2t_{c}} \right]+\frac{P_{Aq}}{\left( b-a \right)t}\left[ \frac{t^{2}+2tt_{c}}{4{t_{c}}^{2}} \right]-\frac{P_{Bq}}{\left( b-a \right)t}\left[ \frac{t^{2}+4tt_{c}}{4{t_{c}}^{2}} \right]+\frac{\left( P_{Bq}-P_{Aq} \right)^{2}}{\left( b-a \right)^{3}t^{2}}\left[ \frac{t}{2t_{c}} \right]^{3}$$

With $\alpha=\frac{t}{2t_{c}}$, we rewrite as:

$$\frac{\partial\Pi_{Bq}}{\partial P_{Bq}}=\frac{2-a-b}{2}+\frac{\alpha\left( b-a \right)}{4}+P_{Aq}\frac{\alpha\left( \alpha+1 \right)}{\left( b-a \right) t}-P_{Bq}\frac{\alpha\left( \alpha+2 \right)}{\left( b-a \right) t}+\left( P_{Bq}-P_{Aq} \right)^{2}\frac{\alpha^{3}}{\left( b-a \right)^{3}t^{2}}$$

$\frac{\partial\Pi_{Bq}}{\partial P_{Bq}}=0\Rightarrow\frac{2-a-b}{2}+\frac{\alpha\left( b-a \right)}{4}=-P_{Aq}\frac{\alpha\left( \alpha+1 \right)}{\left( b-a \right) t}+P_{Bq}\frac{\alpha\left( \alpha+2 \right)}{\left( b-a \right) t}-\left( P_{Bq}-P_{Aq} \right)^{2}\frac{\alpha^{3}}{\left( b-a \right)^{3}t^{2}}$ (9)

**Appendix B.2:**

For firm A the profit is:

$$\Pi_{Aq}=P_{Aq}x_{I}-t\left( \frac{\left( x_{I}-a \right)^{3}+a^{3}}{3} \right)=P_{Aq}x_{I}-t\left( \frac{{x_{I}}^{3}}{3}-{x_{I}}^{2}a+x_{I}a^{2} \right)$$

Therefore, the derivative with respect to the location $a$ translates to:

$$\frac{\partial\Pi_{Aq}}{\partial a}=\frac{\partial P_{Aq}}{\partial a}x_{I}+\frac{1+2\alpha}{6+4\alpha}P_{Aq}-t\left( \frac{1+2\alpha}{6+4\alpha}{x_{I}}^{2}-{x_{I}}^{2}-\frac{1+2\alpha}{6+4\alpha}{2x}_{I}a+\frac{1+2\alpha}{6+4\alpha}a^{2}+2a x_{I} \right)$$

From in $P_{aq}$ (10), we obtain:

$\frac{\partial P_{Aq}}{\partial a}=\frac{t}{4\alpha{(3+2\alpha)}^{2}}\{-4\left( 3+7\alpha+2\alpha^{2} \right)+2a\left( -6+9\alpha+12\alpha^{2}+4\alpha^{3} \right)- 2b\left( 5\alpha+12\alpha^{2}+4\alpha^{3} \right)\}$

Therefore, the derivative becomes:

$$\frac{\partial\Pi_{Bq}}{\partial a}=t\left\{ \frac{-\left( 3+4\alpha\right)}{\alpha\left( 3+2\alpha\right)^{3}}-a\frac{\left( 6+21\alpha+20\alpha^{2}+4\alpha^{3} \right)}{\alpha\left( 3+2\alpha\right)^{3}}-b\frac{2\left( 1+2\alpha\right)}{\left( 3+2\alpha\right)^{3}}-a^{2}\frac{\left( 1+2\alpha\right)^{2}\left( 9+14\alpha+4\alpha^{2} \right)}{4\alpha\left( 3+2\alpha\right)^{3}}-ab\frac{\left( 3+\alpha\right){(\left( 1+2\alpha\right)}^{3}}{2\alpha\left( 3+2\alpha\right)^{3}}+b^{2}\frac{3\left( 1+2\alpha\right)^{2}}{4\alpha\left( 3+2\alpha\right)^{3}} \right\}$$

For firm B the profits are:

$$\Pi_{Bq}=P_{Bq}\left( 1-x_{I} \right)-t\left( \frac{\left( 1-b \right)^{3}+\left( {b-x}_{I} \right)^{3}}{3} \right)$$

$$=P_{Bq}\left( 1-x_{I} \right)-t\left( \frac{{{1-x}_{I}}^{3}}{3}-b+b^{2}-b^{2}x_{I}+b{x_{I}}^{2} \right)$$

Therefore, the derivative with respect to the location $b$ translates to:

$$\frac{\partial\Pi_{Bq}}{\partial b}=\frac{\partial P_{Bq}}{\partial b}\left( 1-x_{I} \right)-\frac{1+2\alpha}{6+4\alpha}P_{Bq}-t\left( -\frac{1+2\alpha}{6+4\alpha}{x_{I}}^{2}-1+2b-2 b x_{I}-b^{2}\frac{1+2\alpha}{6+4\alpha}+{x_{I}}^{2}+2b\frac{1+2\alpha}{6+4\alpha} x_{I} \right)$$

$$\frac{\partial\Pi_{Bq}}{\partial b}=\frac{\partial P_{Bq}}{\partial b}\left( 1-x_{I} \right)-\frac{1+2\alpha}{6+4\alpha}P_{Bq}-t\left( \frac{5+2\alpha}{6+4\alpha}{x_{I}}^{2}-1+2b-2 b\frac{5+2\alpha}{6+4\alpha} x_{I}-b^{2}\frac{1+2\alpha}{6+4\alpha} \right)$$

From in $P_{Bq}$ (11), we obtain:

$$\frac{\partial P_{Bq}}{\partial b}=t\left\{ \frac{6+5\alpha+2\alpha^{2}}{\alpha\left( 3+2\alpha\right)^{2}}-a \frac{\left( 5\alpha+12\alpha^{2}+4\alpha^{3} \right)}{2\alpha{(3+2\alpha)}^{2}} +b\frac{\left( -6+9\alpha+12\alpha^{2}+4\alpha^{3} \right)}{2\alpha{(3+2\alpha)}^{2}} \right\}$$

Therefore, after replacing for $x_{I}$ and $P_{bq}/\partial b$ , the derivative becomes:

$$\frac{\partial\Pi_{Bq}}{\partial b}=\frac{t}{2\alpha}\left( \frac{4\left( 6+23\alpha+33\alpha^{2}+20\alpha^{3}+4\alpha^{4} \right)}{\left( 3+2\alpha\right)^{3}}-a\frac{\alpha\left( 11+38\alpha+36\alpha^{2}+8\alpha^{3} \right)}{\left( 3+2\alpha\right)^{3}}-b\frac{\left( 24+111\alpha+178\alpha^{2}+116\alpha^{3}+24\alpha^{4} \right)}{\left( 3+2\alpha\right)^{3}}-a^{2}\frac{3\left( 1+2\alpha\right)^{2}}{2\left( 3+2\alpha\right)^{3}}+2ab\frac{\left( 3+\alpha\right)\left( 1+2\alpha\right)^{3}}{2\left( 3+2\alpha\right)^{3}}+b^{2}\frac{\left( 1+2\alpha\right)^{2}\left( 9+14\alpha+4\alpha^{2} \right)}{2\left( 3+2\alpha\right)^{3}} \right)$$

**Appendix B.3:**

1. At a=0, the first derivative of the profit function for firm A (Equation.13) becomes:

$$\left. \frac{\partial\Pi_{Aq}}{\partial a} \right|_{a=0}=t\left\{ \frac{-\left( 3+4\alpha\right)}{\alpha\left( 3+2\alpha\right)^{3}}-b\frac{2\left( 1+2\alpha\right)}{\left( 3+2\alpha\right)^{3}}+b^{2}\frac{3\left( 1+2\alpha\right)^{2}}{4\alpha\left( 3+2\alpha\right)^{3}} \right\}$$

$$\left. \frac{\partial\Pi_{Aq}}{\partial a} \right|_{a=0}=-\frac{t(2+b+2b\alpha)(6-3b+\alpha(8-6b))}{4\alpha{(3+2\alpha)}^{3}}$$

1. Since b$\leq1$, this implies that the first derivative of the profit function for firm A is negative at the origin. From (13), we deduce the second derivative of the profit function for firm A is:

$$\frac{{\partial^{2}\Pi}_{Aq}}{\partial^{2}a}=\frac{t}{2\alpha}\left\{ -\frac{2\left( 6+21\alpha+20\alpha^{2}+4\alpha^{3} \right)}{\left( 3+2\alpha\right)^{3}}-a\frac{\left( 1+2\alpha\right)^{2}\left( 9+14\alpha+4\alpha^{2} \right)}{\left( 3+2\alpha\right)^{3}}-b\frac{\left( 3+\alpha\right){(\left( 1+2\alpha\right)}^{3}}{\left( 3+2\alpha\right)^{3}} \right\}$$

Therefore $\frac{{\partial^{2}\Pi}_{a}}{\partial^{2}a}$ is negative everywhere.

1. At b=1, the first derivative of the profit function for firm B (Equation.14) becomes:

$$\left. \frac{\partial\Pi_{Bq}}{\partial b} \right|_{b=1}=\frac{t}{2\alpha}\left( \frac{4\left( 6+23\alpha+33\alpha^{2}+20\alpha^{3}+4\alpha^{4} \right)}{\left( 3+2\alpha\right)^{3}}-a\frac{\alpha\left( 11+38\alpha+36\alpha^{2}+8\alpha^{3} \right)}{\left( 3+2\alpha\right)^{3}}-\frac{\left( 24+111\alpha+178\alpha^{2}+116\alpha^{3}+24\alpha^{4} \right)}{\left( 3+2\alpha\right)^{3}}-a^{2}\frac{3\left( 1+2\alpha\right)^{2}}{2\left( 3+2\alpha\right)^{3}}+2a\frac{\left( 3+\alpha\right)\left( 1+2\alpha\right)^{3}}{2\left( 3+2\alpha\right)^{3}}+\frac{\left( 1+2\alpha\right)^{2}\left( 9+14\alpha+4\alpha^{2} \right)}{2\left( 3+2\alpha\right)^{3}} \right)$$

$$\left. \frac{\partial\Pi_{Bq}}{\partial b} \right|_{b=1}=t\frac{\left( 3-a+2\alpha\left( 1-a \right) \right)\left( 3+2\alpha+a\left( 3+6\alpha\right) \right)}{4\alpha\left( 3+2\alpha\right)^{3}}$$

Since a$\leq1$, this implies that the first derivative of firm B’s profit function is positive at b=1.

1. The second derivative of firm B’s profit function (Equation.14) is:

$$\frac{{\partial^{2}\Pi}_{Bq}}{\partial^{2}b}=\frac{t}{2\alpha}\left\{ -\frac{\left( 24+111\alpha+178\alpha^{2}+116\alpha^{3}+24\alpha^{4} \right)}{\left( 3+2\alpha\right)^{3}}+a\frac{\left( 3+\alpha\right)\left( 1+2\alpha\right)^{3}}{\left( 3+2\alpha\right)^{3}}+b\frac{\left( 1+2\alpha\right)^{2}\left( 9+14\alpha+4\alpha^{2} \right)}{\left( 3+2\alpha\right)^{3}} \right\}$$

Since a≤1 and $b\leq1$, we conclude that:

$$\frac{{\partial^{2}\Pi}_{Bq}}{\partial^{2}b}\leq\frac{t}{2\alpha}\left\{ -\frac{\left( 24+111\alpha+178\alpha^{2}+116\alpha^{3}+24\alpha^{4} \right)}{\left( 3+2\alpha\right)^{3}}+\frac{\left( 3+\alpha\right)\left( 1+2\alpha\right)^{3}}{\left( 3+2\alpha\right)^{3}}+\frac{\left( 1+2\alpha\right)^{2}\left( 9+14\alpha+4\alpha^{2} \right)}{\left( 3+2\alpha\right)^{3}} \right\}$$

$$\frac{{\partial^{2}\Pi}_{Bq}}{\partial^{2}b}\leq\frac{t}{2\alpha}\left\{ -\frac{2(6+21\alpha+20\alpha^{2}+4\alpha^{3})}{{(3+2\alpha)}^{3}} \right\}$$

Therefore $\frac{{\partial^{2}\Pi}_{b}}{\partial^{2}b}$ is negative everywhere.

1. We need to check whether the solutions above are perfect Nash equilibria in prices and locations subgames. This is equivalent to checking whether the optimal profit of firm A ($\Pi_{A})$assuming no undercutting is higher than firm A profit ($\Pi_{Au})$with an undercutting price PAmin and assuming firm B stills charges it is optimal price in the game without undercutting from firm A. We have:

$$P_{Amin}=P_{B}-t_{c}(b-a)(2-a-b)$$

Therefore, we need:

$$\Pi_{A}>\Pi_{Au}, or$$

$$P_{A}*X_{I}-t\int_{0}^{1/2} {(x-a)}^{2}dx>\left( P_{B}-t_{c}\left( b-a \right)\left( 2-a-b \right) \right)*1-t\int_{0}^{1} \left( a-x \right)^{2}dx$$

We check the inequality above for the cases of interior solutions. It is easy to check that for interior solutions (a+b=1), we have:

$P_{A}=P_{B}=\frac{t_{c}}{2}\left\{ (2+\alpha(b-a))\left( b-a \right) \right\}, x_{I}=\frac{1}{2}$, and $P_{Amin}=\alpha\frac{t_{c}}{2}{(b-a)}^{2}$.

$$\Pi_{Au}=\left( P_{B}-t_{c}\left( b-a \right)\left( 2-a-b \right) \right)*1-t\int_{0}^{1} \left( a-x \right)^{2}dx=\alpha\frac{t_{c}}{2}{(1-2a)}^{2}-\frac{t}{3}(3a^{2}-3a+1)$$

$$\Pi_{Au}=\alpha\frac{t_{c}}{2}\left( 1-2a \right)^{2}-2\alpha\frac{t_{c}}{3}\left( 3a^{2}-3a+1 \right)=\alpha\frac{t_{c}}{6}\left( 3-12a+12a^{2}-12a^{2}+12a-4 \right), or$$

$$\Pi_{Au}=-\alpha\frac{t_{c}}{6}$$

However, since the only interior solution is for a=0, the equilibrium is a Nash equilibrium in the prices and locations if $0\leq\alpha<4.$

**Appendix C – The Mixed Model**

**Appendix C.1:**

The profit of firm A is:

$$\Pi_{Am} =P_{Am}x_{Im}-t\int_{0}^{a} \left( a-x \right)dx-t\int_{a}^{x_{lm}} (x-a)dx$$

=$P_{Am}x_{Im}-t\frac{a^{2}}{2}-t\frac{\left( x_{lm}-a \right)^{2}}{2}$

$$\frac{\partial\Pi_{Am}}{\partial P_{Am}}=\frac{P_{Bm}-P_{Am}}{2 \left( b-a \right)t_{c}}+\frac{a+b}{2}-\frac{P_{Am}}{2 \left( b-a \right)t_{c}}+\frac{t}{2 \left( b-a \right)t_{c}}\left[ \frac{P_{Bm}-P_{Am}}{2 \left( b-a \right) t_{c}}+\frac{b-a}{2} \right]$$

$$\frac{\partial\Pi_{Am}}{\partial P_{Am}}=\frac{t}{4t_{c}}+\frac{a+b}{2}+\frac{P_{Bm}-2 P_{Am}}{2 \left( b-a \right)t_{c}}+\frac{t}{2 \left( b-a \right)t_{c}}\left[ \frac{P_{Bm}-P_{Am}}{2 \left( b-a \right) t_{c}} \right]$$

Given $\alpha=\frac{t}{2t_{c}}$. We get:

$$\frac{\partial\Pi_{Am}}{\partial P_{Am}}=\frac{\alpha}{2}+\frac{a+b}{2}+\frac{P_{Bm}-2 P_{Am}}{2 \left( b-a \right)t_{c}}+\alpha\left[ \frac{P_{Bm}-P_{Am}}{{2 \left( b-a \right)}^{2} t_{c}} \right]$$

$\frac{\partial\Pi_{Am}}{\partial P_{Am}}=0\Rightarrow\frac{\alpha}{2}+\frac{a+b}{2} = - \frac{P_{Bm}-2 P_{Am}}{2 \left( b-a \right)t_{c}} - \alpha\left[ \frac{P_{Bm}-P_{Am}}{{2 \left( b-a \right)}^{2} t_{c}} \right]$

The profit of firm B is:

$\Pi_{Bm} =P_{Bm}{(1-x}_{Im})-t\int_{x_{Im}}^{b} \left( b-x \right)dx-t\int_{b}^{1} (x-b)dx$

= $P_{Bm}{(1-x}_{Im})-t \frac{\left( b-x_{Im} \right)^{2}}{2}-t \frac{\left( 1-b \right)^{2}}{2}$

Therefore, the derivative with regard to the price $P_{Bm}$ is:

$$\frac{\partial\Pi_{Bm}}{\partial P_{Bm}}= - \frac{P_{Bm}}{2 \left( b-a \right)t_{c}} + \left[ 1-\frac{P_{Bm}-P_{Am}}{2 \left( b-a \right)t_{c}}-\frac{a+b}{2} \right] -t \left[ \frac{-1}{2 \left( b-a \right)t_{c}} \right] \left[ b-\frac{P_{Bm}-P_{Am}}{2 \left( b-a \right) t_{c}}-\frac{a+b}{2} \right]$$

$$\frac{\partial\Pi_{Bm}}{\partial P_{Bm}}= \frac{2-a-b}{2} +t \left[ \frac{1}{2 \left( b-a \right)t_{c}} \right]\left[ \frac{b-a}{2} \right]- \frac{P_{Bm}}{2 \left( b-a \right)t_{c}}-\frac{P_{Bm}-P_{Am}}{2 \left( b-a \right)t_{c}} -t \left[ \frac{-1}{2 \left( b-a \right)t_{c}} \right]\left[ -\frac{P_{Bm}-P_{Am}}{2 \left( b-a \right) t_{c}} \right]$$

$$\frac{\partial\Pi_{Bm}}{\partial P_{Bm}}= \frac{2-a-b}{2} +\frac{t}{4t_{c}}-\frac{{2 P}_{Bm}-P_{Am}}{2 \left( b-a \right)t_{c}} -\left[ \frac{t}{2 t_{c}} \right]\left[ \frac{P_{Bm}-P_{Am}}{{2 \left( b-a \right)}^{2} t_{c}} \right]$$

$$\frac{\partial\Pi_{Bm}}{\partial P_{Bm}}= \frac{2-a-b}{2} + \frac{\alpha}{2} - \frac{{2 P}_{Bm}-P_{Am}}{2t_{c} \left( b-a \right)} -\alpha\left[ \frac{P_{Bm}-P_{Am}}{{2 t_{c} \left( b-a \right)}^{2}} \right]$$

$$\frac{\partial\Pi_{Bm}}{\partial P_{Bm}}=0\Rightarrow\frac{2-a-b}{2} + \frac{\alpha}{2} = \frac{{2 P}_{Bm}-P_{Am}}{2t_{c} \left( b-a \right)} +\alpha\left[ \frac{P_{Bm}-P_{Am}}{{2 t_{c} \left( b-a \right)}^{2}} \right]$$

**Appendix C.2:**

1. For firm A the profits are:

$$\Pi_{Am}=P_{Am}x_{Im}-t\frac{a^{2}}{2}-t\frac{\left( x_{lm}-a \right)^{2}}{2}=P_{Am}x_{Im}-t\frac{a^{2}}{2}-t\left( \frac{{x_{Im}}^{2}}{2}-x_{Im}a+\frac{a^{2}}{2} \right)$$

$=P_{Am}x_{Im}-ta^{2}-t\frac{{x_{Im}}^{2}}{2}-t x_{Im}a$

Therefore, the derivative with regard to the location $a$ translates as:

$$\frac{\partial\Pi_{Am}}{\partial a}=\frac{\partial P_{Am}}{\partial a}x_{Im}+\frac{\partial x_{Im}}{\partial a}P_{Am}-2 t a-t x_{Im}\frac{\partial x_{Im}}{\partial a} -t x_{Im}-t a\frac{\partial x_{Im}}{\partial a}$$

From (15):

$\frac{\partial P_{Am}}{\partial a}=-\frac{t}{2\alpha{(-3a+3b+2\alpha)}^{2}}\left\{ 6a^{3}+6ab^{2}+4\alpha^{2}\left( 1+\alpha\right)+3a^{2}\left( 2-4b+\alpha\right)-4a\alpha\left( 2+3\alpha\right)+4b\alpha\left( 2+3\alpha\right)-2ab\left( 6+7\alpha\right)+b^{2}\left( 6+11\alpha\right) \right\}$

Also, from (17):

$\frac{\partial x_{Im}}{\partial a}=\frac{1}{2\left( -3a+3b+2\alpha\right)^{2}}\left\{ 3 \left( a-b \right)^{2}-4 \left( 1+a-3b \right) \alpha+4 \alpha^{2} \right\}$

Therefore, the derivative becomes:

$$\frac{\partial\Pi_{Am}}{\partial a}=\frac{t}{4{(-3a+3b+2\alpha)}^{3}\alpha}\left\{ -3 \left( b-a \right)^{3}\left( 2+3a-b \right)\left( 2+a+b \right)-\left( b-a \right)^{2}\left( 24+62a-179a^{2}-14b+228ab-25b^{2} \right)\alpha-2\left( b-a \right)\left( 4+30a-189a^{2}-2b+216ab-27b^{2} \right)\alpha^{2}-4\left( 4a-63a^{2}+72ab-9b^{2} \right)\alpha^{3}-8\left( 7a-b \right)\alpha^{4} \right\}$$

For firm B the profits are:

$$\Pi_{Bm}=P_{Bm}\left( 1-x_{Im} \right)-t\frac{\left( 1-b \right)^{2}}{2}-t\frac{\left( {b-x}_{Im} \right)^{2}}{2}$$

$$=P_{Bm}\left( 1-x_{Im} \right)-\frac{t}{2}+t b-t\frac{b^{2}}{2}-t\frac{b^{2}}{2}-t {b x}_{Im}-t\frac{{x_{Im}}^{2}}{2}$$

Therefore, the derivative with regard to the location $b$ translates as:

$$\frac{\partial\Pi_{Bm}}{\partial b}=\frac{\partial P_{Bq}}{\partial b}\left( 1-x_{Im} \right)-\frac{\partial x_{Im}}{\partial b}P_{Bq}+t-2 t b-t x_{Im}-t b \frac{\partial x_{Im}}{\partial b}-t x_{Im} \frac{\partial x_{Im}}{\partial b}$$

From in $P_{Bm}(16)$, we obtain:

$\frac{\partial P_{Bm}}{\partial b}=\frac{t}{2\alpha{(-3a+3b+2\alpha)}^{2}}\left\{ -6b^{3}+4 \alpha^{2}(1+\alpha)+3 b^{2}(4+\alpha)+4 b \alpha(4+3\alpha)+a^{2}(12-6b+11\alpha)+2a(6b^{2}-2\alpha(4+3\alpha)-b(12+7\alpha)) \right\}$

From in $x_{Im}$, we obtain:

$\frac{\partial x_{Im}}{\partial b}=\frac{1}{2\left( -3a+3b+2\alpha\right)^{2}}\left\{ 3a^{2}+3b^{2}+4b\alpha+4\alpha(1+\alpha)-6a(b+2\alpha) \right\}$

Therefore, after replacing for $x_{I}$ and $P_{bq}/\partial b$ , the derivative becomes:

$$\frac{\partial\Pi_{Bm}}{\partial b}=\frac{t}{4 \alpha\left( 2\alpha+3b-3a \right)^{3}}\left\{ 3\left( 4-a-b \right)\left( 4+a-3b \right)\left( b-a \right)^{3}+\left( a-b \right)^{2}\left( 96-164a-25a^{2}+68b+228ab-179b^{2} \right)\alpha+2\left( b-a \right)\left( 32-160a-27a^{2}+132b+216ab-189b^{2} \right)\alpha^{2}+4\left( 4-54a-9a^{2}+50b+72ab-63b^{2} \right)\alpha^{3}+8\left( 6+a-7b \right)\alpha^{4} \right\}$$

The solutions:

$$a_{1}=\frac{1+17 \alpha+8 \alpha^{2}-\sqrt{9+58 \alpha+145\alpha^{2}+176 \alpha^{3}+64 \alpha^{4}}}{8+48 \alpha}$$

$$b_{1}=\frac{7+31 \alpha-8 \alpha^{2}+\sqrt{9+58 \alpha+145\alpha^{2}+176 \alpha^{3}+64 \alpha^{4}}}{8+48 \alpha}$$

$$a_{2}=\frac{1+17 \alpha+8 \alpha^{2}+\sqrt{9+58 \alpha+145\alpha^{2}+176 \alpha^{3}+64 \alpha^{4}}}{8+48 \alpha}$$

$$b_{2}=\frac{7+31 \alpha-8 \alpha^{2}-\sqrt{9+58 \alpha+145\alpha^{2}+176 \alpha^{3}+64 \alpha^{4}}}{8+48 \alpha}$$

We exclude (a2, b2) since $b_{2}<a_{2}.$

a1 can be written as:

$$a_{1}=f\left( \alpha\right)=\frac{-1+3\alpha+2\alpha^{2}}{[1+17 \alpha+8 \alpha^{2}+\sqrt{9+58 \alpha+145\alpha^{2}+176 \alpha^{3}+64 \alpha^{4}}}$$

1. f is positive and increasing for $\alpha>\alpha_{c}=\frac{1}{4}(-3+\sqrt{17})\simeq0.281$ (See Figure 1). It converges to $\frac{1}{8}$ when $\alpha$ goes to infinity.

As such, for $\alpha>\alpha_{c}$, a1 is an interior solution.


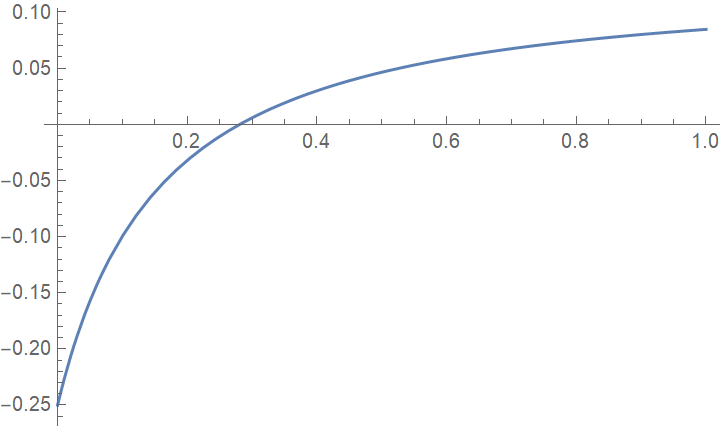


Figure.1. a1 as a function of alpha

1. We need to check whether the solutions above are perfect Nash equilibria in prices and locations subgames. This is equivalent to checking whether the optimal profit of firm A ($\Pi_{Am})$assuming no undercutting is higher than firm A profit ($\Pi_{Au})$with an undercutting price PAmin and assuming firm B stills charges it is optimal price in the game without undercutting from firm A. We have:

$$P_{Am,min}=P_{Bm}-t_{c}(b-a)(2-a-b)$$

Therefore, we need:

$$\Pi_{A}>\Pi_{Au}, or$$

$$P_{A}*X_{I}-ta^{2}-\frac{t}{8}+a\frac{t}{2}>\left( P_{B}-t_{c}\left( b-a \right) \right)*1-t\int_{0}^{a} \left( a-x \right)dx-t\int_{a}^{1} \left( x-a \right)dx$$

We check the inequality above for the cases of interior solutions. It is easy to check that for interior solutions (a+b=1), we have:

$$P_{Am}=P_{Bm}=t_{c}(1-2a)(1+\alpha), x_{I}=\frac{1}{2}$$

$\Pi_{Am}=P_{Am}*X_{I}-ta^{2}-\frac{t}{8}+a\frac{t}{2}=\frac{t_{c}}{2}\left( 1-2a \right)\left( 1+\alpha\right)-ta^{2}-\frac{t}{8}+a\frac{t}{2}=\frac{t_{c}}{2}\left( 1-2a \right)\left( 1+\alpha\right)-2\alpha t_{c}a^{2}-\alpha\frac{t_{c}}{4}+a\alpha t_{c}$

*Therefore,*

$$\Pi_{Am}=\left( \frac{1}{2}+\frac{\alpha}{4} \right)-a-2\alpha a^{2}=g(a)$$

g is continuous and decreasing in a and g(0) is positive and g(1/8) is positive. As such. For all interior solutions defined above, $\Pi_{Am}$is positive.

$$\Pi_{Au}=\left( P_{B}-t_{c}\left( b-a \right) \right)*1-t\int_{0}^{a} \left( a-x \right)dx-t\int_{a}^{1} \left( x-a \right)dx=\left( P_{B}-t_{c}\left( b-a \right) \right)-\frac{ta^{2}}{2}-\frac{t}{2}-\frac{ta^{2}}{2}+ta$$

$$\Pi_{Au}=t_{c}(1-2a)(1+\alpha)-{(1-2a)(t}_{c}+\frac{t}{2})-ta^{2}=t_{c}(1-2a)((1+\alpha)-(1+\alpha))-ta^{2}$$

Therefore,

$$\Pi_{Au}=-ta^{2}$$

Since the profit with undercutting is negative, all the interior solutions, defined in Appendix.5.i (and ii) are Nash equilibrium in the prices and locations for $\alpha.$
